# Medical Device Apps: An Introduction to Regulatory Affairs for Developers

**DOI:** 10.2196/17567

**Published:** 2020-06-26

**Authors:** Lina Keutzer, Ulrika SH Simonsson

**Affiliations:** 1 Department of Pharmaceutical Biosciences Uppsala University Uppsala Sweden

**Keywords:** MDR, medical device regulation, medical devices, medical device software, mHealth, eHealth, mobile apps, smartphone apps

## Abstract

The Poly Implant Prothèse (PIP) scandal in France prompted a revision of the regulations regarding the marketing of medical devices. The new Medical Device Regulation (MDR [EU]) 2017/745 was developed and entered into force on May 25, 2017. After a transition period of 3 years, the regulations must be implemented in all EU and European Economic Area member states. The implementation of this regulation bears many changes for medical device development and marketing, including medical device software and mobile apps. Medical device development and marketing is a complex process by which manufacturers must keep many regulatory requirements and obligations in mind. The objective of this paper is to provide an introduction and overview of regulatory affairs for manufacturers that are new to the field of medical device software and apps with a specific focus on the new MDR, accompanying harmonized standards, and guidance documents from the European Commission. This work provides a concise overview of the qualification and classification of medical device software and apps, conformity assessment routes, technical documentation, clinical evaluation, the involvement of notified bodies, and the unique device identifier. Compared to the previous Medical Device Directive (MDD) 93/42/EEC, the MDR provides greater detail about the requirements for software qualification and classification. In particular, rule 11 sets specific rules for the classification of medical device software and will be described in this paper. In comparison to the previous MDD, the MDR is more stringent, especially regarding the classification of health apps and software. The implementation of the MDR in May 2020 and its interpretation by the authorities will demonstrate how app and software manufacturers as well as patients will be affected by the regulation.

## Introduction

Due to safety issues in the field of medical devices, and especially after the Poly Implant Prothése (PIP) scandal in France, the Medical Device Directive (MDD) 93/42/EEC [1] was revised and replaced with the new Medical Device Regulation (MDR [EU]) 2017/745 [2,3]. The MDR entered into force on May 25, 2017 and must be implemented within the European Union (EU) and European Economic Area (EEA) states after 3 years, by May 26, 2020 [2]. Since the MDR is a regulation, it is immediately enforceable as law in all member states after its implementation date. This contrasts with the previous MDD, which was a directive that member states transpose into national law within a set timeframe [4].

Unlike the US Food & Drug Administration, which regulates foods, medicines, and medical devices, the European Medicines Agency (EMA) regulates only drugs. There is no regulatory body like the EMA for the review and approval of medical devices. Manufacturers themselves declare conformity of their devices with the European legislations and regulations and affix a CE (Communauté europeénne) mark (Article 10 and 20 MDR). Products that bear a CE mark can then be marketed within the EU/EEA (Articles 2 and 10 MDR). Affixing the CE mark to a product is only legal after a conformity assessment has been performed (Article 20 MDR). Depending on the class of the device, a notified body must be involved in this process (Introduction [60] and Articles 52-53 MDR). For certain highly critical or novel products, an additional examination by so called “expert panels” is mandatory (Introduction [56] and Article 106 MDR).

Independent of the risk class, technical documentation (TD) must be compiled to allow an assessment of whether the general safety and performance requirements set by the MDR are met (Annex II MDR). With the exception of class l devices, the notified bodies then inspect the manufacturer’s Quality Management System (QMS) and technical documentation and subsequently issue the required Annex certificates (Annex XII MDR). These are prerequisites for declaring conformity and affixing the CE mark (Article 10[6] MDR). For guidance on how to perform the steps required for CE marking, including risk management, technical documentation, and QMS, and to prove regulatory compliance, manufacturers are advised to work according to harmonized standards and common specifications (Articles 8-9 and Annex II [4c] MDR). When the MDR came into force in 2017, there was no associated harmonized standard or common specification. According to the European Commission, these will be implemented soon [5].

**Figure 1:**
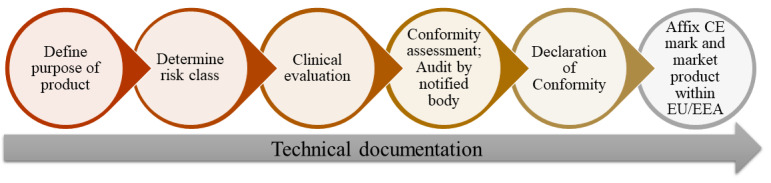
Important stages of medical device development.

When developers of software or mobile apps claim that their product has a medical purpose, it becomes a medical device and must bear a CE mark (Article 2[1] MDR). This paper describes the process of placing mobile apps and software on the market as medical devices (Figure 1) and serves as an introduction to regulatory affairs for app and software developers.

## Elements of the Medical Device Regulation

The primary elements of the new Medical Device Regulation (MDR [EU]) 2017/745 and the accompanying harmonized standards and guidance documents provided by the European Commission are described below.

### Qualification of Mobile Apps and Software: What Constitutes a Medical Device?

#### Medical Device Software

Mobile apps and software that are independent of any device and are not intended to be used as an accessory to a medical device are referred to as Medical Device Software (MDSW) or standalone software [6] and must be qualified and classified in their own right (Annex VIII [3.3] MDR).

#### Intended Purpose

The first, essential question an app or software developer must answer is whether the product is a medical device or not. Software qualifies as a medical device if the developer’s stated purpose of the software meets the definition of a medical device in Article 2[1] of the MDR (Textbox 1).

**Medical device definition [Article 2(1) MDR].**

“Medical device” means any instrument, apparatus, appliance, software, implant, reagent, material or other article intended by the manufacturer to be used, alone or in combination, for human beings for one or more of the following specific medical purposes:

- diagnosis, prevention, monitoring, prediction, prognosis, treatment or alleviation of disease,
- diagnosis, monitoring, treatment, alleviation of, or compensation for, an injury or disability,
- investigation, replacement or modification of the anatomy or of a physiological or pathological process or state,
- providing information by means of in vitro examination of specimens derived from the human body, including organ, blood and tissue donations, and which does not achieve its principal intended action by pharmacological, immunological or metabolic means, in or on the human body, but which may be assisted in its function by such means. The following products shall also be deemed to be medical devices:
  - devices for the control or support of conception;
  - products specifically intended for the cleaning, disinfection or sterilisation of devices as referred to in Article 1(4) and of those referred to in the first paragraph of this point.

The decision of whether a software product qualifies as a medical device is made by the developer or, using the terminology of the MDR, the manufacturer (Article 2[30] MDR). If the manufacturer states that the system can be used for a medical purpose, it must be CE marked (Articles 10 and 20 MDR). Whether a product qualifies as a medical device is determined by the intended use, as stated by the manufacturer and the mechanism of action of the product, not the design or user [6]. Furthermore, the description of the intended purpose must include a statement of benefit for the patient, otherwise it cannot be marketed as a medical device (Articles 61-62, Annexes XIV and XV MDR).

#### Key Characteristics for Qualification as a Medical Device

If a mobile app or software performs an action on data beyond storage, archiving, communication, or simple search; the performed action is for medical purposes; and the performed action is for the benefit of an individual patient, it most likely qualifies as a medical device and is subject to the MDR [6]. For further guidance on qualification of standalone software, see “MEDDEV 2.1/6 Guidelines on the qualification and classification of standalone software” [7].

### Classification of Standalone Software Including Apps

The MDR defines four risk classes: I, IIa, IIb, and III (Table 1). For classification of software, rule 11 has been included in the regulation (Textbox 2) (Annex VIII MDR).

**Table 1:** Summary of differences between risk classes.

Class	Documentation	Notified body involved?	QMS^a^	Certificates	Clinical investigation
I (low risk)	Manufacturer must compile the technical documentation and self-declare conformity	No	Yes	No	Not mandatory. May be required depending on the outcome of the clinical evaluation
IIa (low-medium risk)	Manufacturer must draw up the technical documentation and apply to a European Notified Body	Yes	Yes, certified	Yes (Annex IX certificate, QMS certificate)	Not mandatory. May be required depending on the outcome of the clinical evaluation
IIb (medium-high risk)	Manufacturer must draw up the technical documentation and apply to a European Notified Body	Yes	Yes, certified	Yes (Annex IX certificate, QMS certificate)	Not mandatory. May be required depending on the outcome of the clinical evaluation
III (high risk)	Manufacturer must draw up the technical documentation and apply to a European Notified Body	Yes, expert panel	Yes, certified	Yes (Annex IX certificate, QMS certificate)	Mandatory

^a^QMS: Quality Management System.

**Rule 11, Annex VIII MDR.**

Software intended to provide information which is used to take decisions with diagnosis or therapeutic purposes is classified as class IIa, except if such decisions have an impact that may cause:

- Death or an irreversible deterioration of a person's state of health, in which case it is in class III; or
- Serious deterioration of a person's state of health or a surgical intervention, in which case it is classified as class IIb.

Software intended to monitor physiological processes is classified as class IIa, except if it is intended for monitoring of vital physiological parameters, where the nature of variations of those parameters is such that it could result in immediate danger to the patient, in which case it is classified as class IIb. All other software are classified as class I.

This rule implies that many apps might have to be classified as class IIa, IIb, or III in the future, while under the former Medical Device Directive (MDD) 93/42/EEC most standalone software including apps were classified as class l or not designated as medical devices at all [1]. Interpreting rule 11 on its own indicates that, for example, software used to calculate dosages of drugs with high toxicity, suggesting a diagnosis, or aiding with therapy or radiation planning will fall within class III, since a mistake might cause death. If it is highly unlikely that death could be caused by an error, it could fall within class IIb, which is defined as a device for which “…a mistake can cause serious deterioration of a person’s state of health...” (Annex VIII MDR). Medical device software may only fall under class IIa if a mistake cannot be anticipated to cause serious deterioration of a person’s state of health. Medical device software may fall under class I only if it is not intended to provide information used to make decisions for diagnostic or therapeutic purposes. For class I devices, no notified body is involved in the declaration of conformity.

These classification criteria are very stringent and prompted formation of the Medical Device Coordination Group (MDCG), an official working group serving to advise the European Commission regarding medical devices [8]. The group recently released a guidance document that further elaborates rule 11 [6]. The guidance provides examples on how to classify software, and the authors seem to interpret rule 11 less strictly than an original reading of the MDR would suggest. The document suggests for instance that software “intended to rank therapeutic suggestions for a health care professional based on patient history, imaging test results, and patient characteristics…, should be classified as class IIa...”. If one merely reads rule 11, one could conclude that these types of software would fall within class III, since an error might cause death. This guidance is not legally binding [6] but notified bodies might consider it when making their decision.

### Conformity Assessment Routes

Fulfilling the “General safety and performance requirements” described in Annex I of the MDR is the most crucial step on the long road to marketing a medical device. To prove compliance with these requirements, manufacturers must follow one of the conformity assessment procedures described in the MDR appendices. Typically, manufacturers apply harmonized standards, and in the future also common specifications, to prove compliance with these requirements (Articles 8-9 and Annex II [4C] MDR). Examples of general safety and performance requirements include risk management, software lifecycle processes, software verification and validation, and usability (Annex I MDR). A complete list of requirements can be found in Annex I of the MDR.

Conformity assessment is a process demonstrating whether the “general safety and performance requirements” of the MDR (Annex I MDR) have been fulfilled. Once the conformity of the medical device with the requirements has been proven, the manufacturer may declare the conformity, CE mark and market the product within the EU/EEA (Articles 19-20 MDR). Depending on the risk class, the MDR describes three different paths of conformity assessment in accordance with Annexes IX, X and XI (Article 56 MDR). Besides the four main risk classes (I, IIa, IIb, III), the MDR defines three sub-classes for risk class I, which are devices with measuring function (I_m_), sterile devices (I_s_) and reusable surgical instruments (I_r_). For classes I_m_, I_s_, I_r_, IIa, IIb and III, a notified body must be involved in the process of conformity assessment. This is not required for all remaining devices falling within class l (Articles 52-53 MDR). For risk class III, an additional expert panel will scrutinize the clinical evaluation and assess whether the clinical data is sufficient to provide confidence in the safety and performance of the device (Annex IX MDR). For medical device software, the development process cannot be ignored since it is difficult to find errors by simply testing the finished product, which would be the case in the Annex XI (Part B) conformity assessment. In contrast, the procedure described in Annex IX includes an assessment of the QMS and the technical documentation by a notified body. The product of a successful assessment is an Annex IX certificate and an EU QMS certificate for the manufacturer, who can then declare conformity of the medical device with the requirements set by the MDR (Articles 19 and 56 MDR).

### Technical Documentation

According to the “general obligations of manufacturers” (Article 10[4] MDR), technical documentation must be compiled and kept up to date to enable assessment of compliance with the safety and performance requirements set by the MDR. Annex II of the MDR lists in detail what is required in the documentation, including documentation of a fully implemented risk management system, benefit-risk analysis, clinical evaluation report, software life cycle file, usability file, and many other requirements.

### Clinical Evaluation

The supporting documentation for the CE declaration must include a clinical evaluation. This is an evaluation of side effects and the acceptability of the benefit-risk ratio based on clinical data (Article 61 MDR). Conducting a proper clinical evaluation will demonstrate (1) which clinical data are necessary; (2) which clinical data can be adequately supplemented by methods other than clinical investigations, such as published literature, prior clinical investigations, clinical experience, or by using suitable clinical data from equivalent devices; and (3) which clinical data remain to be delivered by clinical investigations (Article 61 MDR). Clinical investigations within the MDR are what many people would refer to as “clinical trials” and are defined as “systematic investigation involving one or more human subjects, undertaken to assess the safety or performance of a device” (Article 2[45] MDR). It is one of the methods to obtain clinical data supporting treatment efficacy and to confirm clinical benefit (Article 62 MDR). If sufficient clinical data to perform a clinical evaluation can be retrieved from the literature or other sources, the manufacturer can proceed without a clinical investigation. The clinical evaluation must be updated frequently with data from post-market surveillance. New data, as well as considerations for new or changed intended purposes, require an updated clinical evaluation and may indicate the necessity for additional clinical investigations (Article 61 MDR).

The clinical evaluation must be planned, conducted, and documented. The clinical evaluation, its results, and the clinical evidence derived from it must be documented in a clinical evaluation report and included as part of the technical documentation (Annexes II and XIV MDR[9]). Furthermore, a clinical evaluation plan must be elaborated and documented in the technical documentation (Annexes II and XIV MDR).

#### Regulatory Requirements for the Clinical Evaluation

App developers are advised to follow the regulations and guidelines listed in Textbox 3.

**Regulations and guidelines related to the clinical evaluation.**

- MDR
  - Chapter ll, Article 10[3]
  - Chapter Vl, Article 61
  - Annex XlV (Part A)
- MEDDEV 2.7/1 revision 4 - Clinical evaluation: Guide for manufacturers and notified bodies
- EN ISO 14155-1
- EN ISO 14155-2

#### When Must a Clinical Investigation Be Undertaken?

A clinical investigation is always mandatory for class III devices, regardless of the amount of information that can be retrieved from other sources (Article 61[4] MDR).

Depending on clinical claims, the outcome of risk management, and the results of the clinical evaluation, clinical investigations may also have to be performed for nonimplantable medical devices classified as I, IIa, and IIb. In addition, a clinical investigation must be conducted if there was no sufficient pre-existing clinical investigation data or scientific literature on which to base a clinical evaluation (Article 61 MDR).

Clinical investigations are only to be performed when the information necessary on device performance, safety, and clinical benefit cannot be obtained in any way other than by testing the device on humans (Articles 61,62 MDR).

#### Notified Bodies

Notified bodies must be involved in the conformity assessment procedures for device classes I_s_, I_m_, I_r_, IIa, Ib and III (Articles 52,53 MDR). A notified body is an organization designated by national authorities to assess the conformity of certain products with the appendices of the MDR, harmonized standards, and common specifications before being placed on the market. Manufacturers can freely choose between notified bodies that have an expertise in the relevant product area [9]. An official list of notified bodies can be found on the European Commission’s website [10].

### What are Common Specifications?

**Figure 2:**
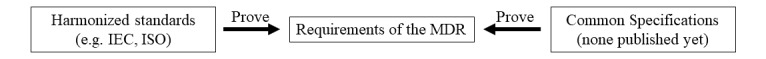
Common specifications and harmonized standards.

The MDR introduces the concept of “Common Specifications” (CS), which are similar to the already existing harmonized standards (Figure 2). In cases where there are no applicable harmonized standards, insufficient harmonized standards, or where there is a need to address public health concerns, common specifications must be followed in order to demonstrate device compliance with the requirements set by the MDR (Article 9 MDR). One should be aware of this concept since according to Article 9 those CS must be followed. Even when there are no CS available, it is important to stay up to date on developments.

### Software Life Cycle

Software development must follow the principles of a software life cycle (Annex l [17.2] MDR). A software life cycle consists of software development and validation/verification, software maintenance, problem resolution, risk management, and configuration management [11]. Compliance with International Standards Organization (ISO) standard IEC 62304 (Medical device software - Software life cycle processes) meets the requirements of the MDR [11].

### Unique Device Identifier

The Unique Device Identifier (UDI) system was first introduced by the US Food and Drug Administration (FDA) [12] and will also apply to the EU market once the MDR is in force (Article 27 and Annex VI MDR). The UDI-number is linked to the European database of medical devices (EUDAMED), contains all relevant information about a medical device, and is used to identify every device (Articles 27 and 33 MDR). This system has been developed to help track each device, react quickly in case of a serious incident, and prevent marketing of illegal devices (Introduction [41] MDR). This should improve vigilance and consequently patient safety [13]. The UDI code must be affixed as a 2D/Data matrix code, inter alia, ID/linear barcode or radio-frequency identification (RFID), and in a human readable interpretation (HRI) format. The manufacturer must place the UDI on every single product. In case of medical device software and apps, the UDI must be stated within the software, such as in the “about” file or the start-up screen. A medical device for clinical investigation must not have a UDI (Annex VI MDR). According to Article 27 of the MDR, the manufacturer shall keep an updated list of all UDIs as part of the technical documentation. The UDI will be assigned by organizations established for this purpose (Article 27 MDR). Further information can be found in Articles 27-28 and Annex VI (Part C) of the MDR. A guidance document on how to create the UDI database and which data format should be used has been provided by the EU UDI Work Group [14].

#### Basic-UDI-DI

The Basic UDI-DI is the primary identifier of a device model in EUDAMED and must be referenced in relevant certificates and the EU declaration of conformity. This number will not be affixed to the product. Similar products with the same purpose, such as those that only differ in user interface language, will carry the same Basic-UDI-DI (Part C of Annex VI MDR).

#### UDI-DI

The UDI-DI is the identifier specific to a device and manufacturer. Software with different user interface languages must carry different UDI-DIs. This part of the UDI is fixed (Part C of Annex VI MDR).

#### UDI-PI

The UDI-PI is the product identifier used to mark a production series of a device (eg, batch, serial number, software identification) and is affixed to every single product. Each software version has its own UDI-PI. This part of the UDI is variable (Part C of Annex VI MDR). For software, not every single installation/download will have its own UDI-PI, but each version of the software does require it.

#### When is a New UDI-DI Needed?

A new UDI-DI is needed in the cases presented in Textbox 4.

**Situations in which a new UDI-DI is needed for apps or medical device software (Part C of Annex VI MDR).**

- Changes in performance, efficacy, safety, intended use of the software or interpretation of data
- Changes in algorithms, database structures, operating platforms, architecture, user interface or channels for interoperability
- Change of the software name
- Change in user interface language

#### When is a new UDI-PI needed?

A new UDI-PI is necessary after minor software revisions such as bug fixes, usability enhancements (those that are not for safety purposes), security patches, or operating efficiency (Part C of Annex VI MDR).

## Conclusion

While the implementation of the new, more stringent MDR might lead to the development of more high-quality apps and improved patient safety, it might also limit the development and release of new apps and software on the market. The classification of a device as class IIa or higher requires evaluation by a notified body, which can be very costly and therefore a barrier to entry for app developers. Whether the new MDR and especially rule 11 is a blessing or a curse for app developers will depend on authorities’ interpretation of the guidelines and can only be evaluated after implementation in May 2020.

## Abbreviations

CE: Communauté europeénne
CS: Common Specifications
EEA: European Economic Area
EMA: European Medicines Agency
EU: European Union
EUDAMED: European Database on Medical Devices
FDA: US Food and Drug Administration
HRI: Human Readable Interpretation
ISO: International Organization for Standardization
MDCG: Medical Device Coordination Group
MDD: Medical Device Directive 93/42/EEC
MDR: Medical Device Regulation (EU) 2017/745
MDSW: Medical Device Software
PIP: Poly Implant Prothése
QMS: Quality Management System
RFID: radio-frequency identification
TD: technical documentation
UDI: Unique Device Identifier
UDI-DI: Unique Device Identifier – Device Identifier
UDI-PI: Unique Device Identifier – Product Identifier
